# Procedures for Breadmaking Quality Assessment of Rye Wholemeal Flour

**DOI:** 10.3390/foods8080331

**Published:** 2019-08-08

**Authors:** Sylwia Stępniewska, Waleed H. Hassoon, Anna Szafrańska, Grażyna Cacak-Pietrzak, Dariusz Dziki

**Affiliations:** 1 Department of Grain Processing and Bakery, Prof. Wacław Dąbrowski Institute of Agricultural and Food Biotechnology, 02-532 Warsaw, Poland; 2 Department of Food Technology, College of Food Sciences, University of Al-Qasim Green, Babylon 964, Iraq; 3 Department of Thermal Technology and Food Process Engineering, University of Life Sciences in Lublin, 1, 20-286 Lublin, Poland; 4 Division of Cereal Technology, Faculty of Food Sciences, Warsaw University of Life Sciences, Nowoursynowska 159C, 02-787 Warsaw, Poland

**Keywords:** wholemeal flour, rye, pentosans, rheological properties, quality

## Abstract

The aim of this study was to evaluate the baking value of rye flours from industrial mills and to indicate which rye flour quality parameters are the most important predictors of wholemeal rye bread quality for commercially milled rye grains. Ten wholemeal rye flours, which were characterized by ash content ranging from 1.43% to 2.42% d.m. (dry mass), were used for the study. The parameters that characterize the flour properties and the baking test were assessed. The study revealed that for the analyzed commercial rye flours, the falling number test and the amylograph properties are insufficient parameters for predicting the quality of wholemeal rye bread. The manufacture of good quality wholemeal bread requires the use of rye flour with superior quality, such as fine granulation, low protein content, low total and insoluble pentosans content, and, in particular, a high percentage of water-soluble pentosans content. Breads with a higher volume were obtained from rye flours that were generally characterized by lower protein content, lower total and insoluble pentosans content, and higher water-soluble pentosans content. Flour granulation and the percentage of water-soluble pentosans content especially, had a significant impact on bread’s hardness of crumb and the hardness of crumb’s increase during bread storage.

## 1. Introduction

Rye (*Secale cereale* L.) is a traditional raw material used for the production of bread in Northern and Eastern Europe. Poland, next to Russia, Germany, Ukraine, and Belarus, is a major producer of rye grain [1]. Starch and pentosans are very important components of rye flour. Pentosans play a main role in developing the dough properties at temperature below 45 °C, while starch has an influence on the structure of crumb when temperature exceeds 45 °C [2]. Rye starch gelatinizes at temperatures of 55–70 °C, which converges with the range of temperature for optimal α-amylase activity [3]. The most adequate tests for evaluating the properties of starch are the falling number test and the amylograph test [4,5]. Except starch, pentosans content (PC) and enzyme activity are important factors for the baking performance of rye flour. These components primarily have the ability to absorb a significant amount of water and affect the increase in dough viscosity [6,7]. During dough development, pentosans disturb the protein network formation and influence on the properties of dough [8,9]. Pentosans are mainly located in the external parts of rye kernel and are classified as water-soluble (WSP) and water-insoluble (WIP) [10,11]. Previous studies have often reported that WSP are beneficial, while WIP have negative effects on the baking quality of rye flour [12,13]. A study by Cyran and Cygankiewicz [14] showed that not only the quantity of pentosans but also the proportion of WSP:WIP contributes to properties of flour and bread properties. The above study revealed that good quality rye flour should be characterized by a high ratio of WSP:WIP. More desirable for breadmaking is rye flour with a high total pentosans’ content, particularly with a higher percentage of water-soluble pentosans’ content [15]. The swelling curve test is used to determine the dough properties associated with PC and the activity of enzymes responsible for degradation of cell wall [16]. There are limited studies on the baking quality of rye flour. Banu [17] studied low-extraction rye flours obtained from milled rye grain in a laboratory mill. Buksa et al. [12], Salmenkallio-Marttila and Hovinen [18] examined wholemeal flours prepared from rye grains in different seasons. However, there are no studies regarding the assessment of baking quality of rye flour obtained from grain in industrial mills. 

Therefore, the objective of this study was to evaluate the quality of high-extraction rye flours from industrial mills, especially in terms of the relationship between flour and bread properties. Furthermore, the aim of this work was to indicate the quality of flour parameters that are the most important predictors of rye bread quality for commercially milled rye grain.

## 2. Material and Methods

### 2.1. Material

Ten wholemeal rye flours (samples from RF1 to RF10), which were characterized by ash content ranging from 1.43% to 2.42% d.m., were used in this study. The rye flours were produced in different commercial mills located in Poland. 

### 2.2. Methods

#### 2.2.1. Tests of Flour Quality

The following quality parameters of the tested samples of rye flours were assessed: Protein content (PC) according to ISO 20483:2013 [19], ash content (AC) according to ISO 2171:2007 [20], the falling number test (FN) according to ISO 3093:2009 [21], (FN informs about the alpha-amylase activity in flour and is defined as the time (in seconds) required to stir and to allow a stirrer to fall down through a hot flour gel), and the amylograph properties, such as amylograph peak viscosity (APV) and final temperature of starch gelatinization (FT) according to ISO 7973:1992 [22]. The flour particle size distribution tests (granulation, Gr) were performed by sifting 100 g of a sample for 3 min, in a laboratory sifter (AS 200, Duesseldorf, Germany) with 95-μm pore size sieves. The total pentosans content (TPC) and water-soluble pentosan content (WSP) were determined [23]. The water-insoluble pentosans content (WIP) was calculated from the difference between TPC and WSP. Water absorption (WA) of the tested rye flours was determined according to Hansen et al. [24] by using a 300 g Farinograph bowl (Brabender, Germany). The final consistency was 300 FU.

#### 2.2.2. Swelling Curve (SC)

The SC test was performed by preparing a suspension from 120 g flour (14% moisture, dry mass), 364 mL of water, and 46 mL of buffer (sodium phosphate buffer solution pH = 5.0), and that suspension was tested in an amylograph (Brabender, Duisburg, Germany) under constant stirring in the temperature range 30 °C to 42 °C. The slurry was then held at 42 °C for 0.5 h [25]. The detected parameters measured in Brabender units (BU) were as follows: Initial viscosity AV (viscosity at 30 °C), viscosity BV (viscosity at 42 °C), and viscosity CV (viscosity after holding suspension at 42 °C over 30 min). The changes in viscosity at 42 °C [log(BV − CV)], which characterize, indirectly, the activity of enzymes that hydrolyze pentosans during the first phase of baking, were calculated with the following formula

(1) log(BV−CV)=(logBV−logCV)×1000

#### 2.2.3. Baking Test

Rye breads were produced in the laboratory baking trials; bread was made with sourdough (SO). Initially, a part of flour was soured in a one-stage process. SO with a yield of 250% was prepared with 350 g of each rye flour (adjusted to standard moisture of 14%), 3.5 g of starter [16], and 525 cm^3^ of water (temperature in the range of 35–36 °C) to obtain a homogeneous dough with soft consistency. SO fermentation was performed for 20 h at 30 °C and 75% RH. The final step was as follows: The mature SO, 650 g of rye four, 10 g of yeast, 17 g of salt, with the water equivalent to farinograph absorption at 300 FU, decreased in absorption by the water added during preparation of the SO. The dough was prepared in the spiral mixer (Turbo-mix-6,5, HOMMEL, Wülfrath, Germany) at a low speed to obtain a homogeneous mass (for approximately 10 min). Dough temperature after mixing was in the range of 30–32 °C. The dough was placed, for 10 min, in the fermentation cabinet. After fermentation, the dough was divided into pieces (5 pieces, each with 350 g weight) and manually molded. The formed pieces were placed in tins and proofed in at 75% relative humidity and 35 °C until the dough surface reached the top of the tin. Baking was performed in a decking oven Piccolo Wachtel Winkel (Hilden, Germany) with steaming at the beginning of the baking (approximately 10 s). During the first 10 min of baking, the temperature was 260 °C, and the temperature was then decreased to 220 °C. After 40 min of baking, the breads were cooled at room temperature, and stored in polyethylene bags.

#### 2.2.4. Bread Characteristics

Rye bread quality was assessed using the following parameters: Bread yield, bread volume, bread crumb hardness, and crumb moisture. Bread property evaluation was performed 20 ± 4 h after baking. The crumb hardness was also determined four days after baking. The bread yield (YB), (the amount of bread obtained from 100 g of flour) was calculated according to [16].

The loaf volume was measured using the millet seed displacement method, and the specific bread volume was expressed as cm^3^/100 g of bread. The bread crumb hardness was determined using the texture analyzer Instron 1140 (Zurich, Switzerland) according to the methodology described by Stępniewska et al. [16]. The moisture of crumb was determined by drying 10 g samples of bread (at 130 °C for 1.5 h). 

#### 2.2.5. Statistical Analysis

One-way analysis of variance (ANOVA) was performed, and the homogenous groups were determined by Tukey’s test. Pearson’s correlation coefficients were calculated (significance level of α was established at 0.05). Principal component analysis (PCA) was also performed. For a better overview, the PCA was performed as in [16]. Moreover, for Pearson’s correlation coefficients, an additional significance level of α = 0.01 was used. Data were analyzed using Statistica 10 software (TIBCO Software, Palo Alto, CA, USA

## 3. Results and Discussion

### 3.1. Flour Properties

The rye flour samples significantly differed according to the PC and AC (Table 1). The PC ranged from 9.2% d.m. (dry mass) (RF4) to 14.0% d.m. (RF10), while the ash content was in the range of 1.43% d.m. (RF4) to 2.42% d.m. (RF10). In the present study, rye flour RF4 was characterized by a significantly lower protein content and ash content than those of the other tested rye flour samples, while the flour samples RF8 and RF10 were characterized by a significantly higher ash content than those of the other tested rye flour samples.

Granulation is a quality parameter that significantly affects the dough behavior during fermentation. On the one hand, fermentation of dough with a finer flour is more intense, which results from easier access of enzymes to starch. On the other hand, a flour with very fine granulation may have higher buffering properties, which is associated with a higher content of soluble ingredients; that poses a risk of excessive degradation of substrates responsible for creating the dough and bread structure [26]. The rye flour samples differed significantly in terms of granulation (Table 1). The flours RF10 and RF8 were characterized by the lowest amount of particles below 95 µm (12.5% and 13.6%, respectively), whereas the sample RF4 was characterized by the highest mass fraction of particles below 95 µm (39.0%).

The rye flour samples differed significantly in terms of water absorption, which was in the range of 54.0% (RF1) to 66.6% (RF10) (Table 1). Because the rye flours were produced in industrial mills, according to different milling technologies, they were probably characterized by different degrees of starch damage, a parameter that could affect the variability of the tested rye flours in terms of water absorption [27]. 

The rye flour samples significantly varied with regard to α-amylase activity and starch properties, such as FN, APV, and FT (Table 1). These parameters provide information on the starch susceptibility to enzyme degradation and ability of starch for gelatinization and swelling [1,4]. The FN changed from 160 s (RF6) to 278 s (RF3). Flours with an FN value from 125 to 200 s are required to produce good quality rye bread [28]. In the present study, only two tested flour samples (RF5 and RF6) were characterized by FNs below 200 s, while the remaining tested rye flour samples showed low α-amylase activity (FN values were above 200 s). That probably means that the dough from those rye flour samples were rigid and firm, and the obtained bread should be characterized by low volume, and a dry, dense, and hard crumb [4,29]. The tested rye flour samples differed significantly according to amylograph properties such as APV and FT (Table 1). APV ranged from 260 AU (RF9) to 480 AU (RF3), while FT ranged from 68.0 °C (RF5) to 83.0 °C (RF8). Rye flour shows optimal bread making quality with an APV in the range of 400–600 AU and with an FT in the range of 65 to 69 °C [29]. In the present study, only three rye flour samples, namely RF3 (480 AU), RF4 (450 AU), and RF8 (430 AU), were characterized by the optimal value of APV. Further, the value of FT for most of the tested rye flour samples was above the optimal range. Among the tested rye flour samples, only two samples (RF5 and RF6) were characterized by the appropriate value of FT.

The results of pentosans content in the tested rye flour samples are presented in Table 2. Statistical analysis revealed that the tested rye flours showed significant differences in terms of pentosans content. 

The total pentosans content (TPC) ranged from 8.3% d.m. (RF9) to 13.4% d.m. (RF10). Generally, rye flours with higher TPC were also characterized by a significantly higher WA. The correlation coefficient between TPC and WA was significant, and equaled 0.868 (Table 3). Hansen et al. [24] showed a positive correlation between TPC and WA. In our study, TPC was more correlated with WA than with PC. That finding confirmed the observation of Banu et al. [30], who found that in rye flour obtained from a laboratory mill, water absorption and holding capacity are influenced more by the concentration of pentosans than by protein quality and quantity. A positive and significant correlation between TPC and AC (*r* = 0.893, Table 3), and a negative correlation between Gr and TPC (*r* = −0.638, Table 3) were also observed. The water-soluble pentosans content (WSP) ranged from 2.4% d.m. (RF2) to 3.9% d.m. (RF10). The correlation between WSP and WA was significant and positive (*r* = 0.639, *p* < 0.05, Table 3). A similar relationship between WSP and WA was found by other authors [27]. A study conducted by Buksa et al. [31,32] revealed that the ability of water soluble pentosans to bind water was significantly influenced, not only by their content, but also by their molecular mass. An increase in the mass of WSP cause an increase in flour water absorption. In the present study, sample RF7 contained significantly higher WSP than flour RF5. However, RF7 was characterized by a significantly lower water absorption. This is probably caused by the differences in the molecular weight of WSP. The water-insoluble pentosans content (WIP) ranged from 5.4% d.m. (RF1) to 9.7% d.m. (RF8). Compared to the other tested flours, flour samples RF10 and RF8 were characterized by a significantly higher WIP. The WIP correlated positively with PC and AC (*r* = 0.768, *r* = 0.864, respectively, Table 3). The percentage of the WSP from the total pentosans content (%WSP) showed a high range of variation, which was in the accordance with the results of Buksa et al. [12]. The flour sample RF2 was characterized by the lowest %WSP (25%), while the sample RF7 was characterized by the highest %WSP (37%).

### 3.2. The Swelling Curve Parameters

The results of the swelling curve test are presented in Table 2. Statistical analyses revealed significant differences between the tested rye flours for all parameters determined on the basis of a swelling curve. The initial viscosity (AV), which is mainly related to the amount of pentosans in rye flour, changed from 100 BU (slurry from the flour RF2) to 670 BU (slurry from the flour RF10), and the viscosity when the sample reached the temperature of 42 °C (BV) changed from 110 BU (slurry from the flour RF2) to 635 BU (slurry from the flour RF10). It was found that AV and BV were positively correlated with AV (*r* = 0.673, *r* = 0.685, respectively, Table 3). The final viscosity (CV), was in the range of 145 BU (slurry from the flour RF2) to 410 BU (slurry from the flour RF10). The CV depends on the amount and hydration properties of pentosans, and the degree of enzymatic degradation. The value of log(BV − CV), which informs on the hydrolysis of pentosans, also showed a large variation between the tested flour samples. The rate at which the viscosity decreases can be an indicator of the decomposition of soluble pentosans—both those present in flour and resulting from the transformation of insoluble pentosans in the first phase of baking [17]. A moderate level of pentosan hydrolysis enhances dough elasticity, which increases the ability to retain CO_2_ by the dough and has positive results for bread volume. However, excessive depolymerization of pentosans decreases this ability, due to low viscosity and thus has a negative effect on bread properties [33]. The values of log(BV − CV) changed from −120 (RF2) to 190 (RF10). The negative value of log(BV − CV) for the flour RF2 indicated very low activity of enzymes that hydrolyze pentosans; this means that during breadmaking, the transformation of WIP into WSP would be hardly possible. The soluble forms of pentosans developed from the transformation of insoluble pentosans were probably characterized by a higher ability to bind water, causing a reduction of water access during gelatinization of starch and stiffening of the dough, which in turn led to obtaining low-volume bread with a dense and hard crumb [34]. It was found that WSP had a positive impact on the value of the logarithmic decrease in viscosity (*r* = 0.769, Table 3). 

### 3.3. Bread Characteristics

The specific bread volume (SBV) ranged from 146 cm^3^/100 g of bread (bread from flour RF10) to 191 cm^3^/100 g of bread (bread from the flour RF9, Table 4). The breads obtained from the rye flour samples RF2 and RF10, characterized by the highest protein content, showed a significantly lower volume than breads obtained from other flour samples. The correlation between SBV and PC was significant, and equal to *r* = −0.689 (Table 5). Further, a positive correlation between SBV and %WSP (*r* = 0.641, Table 5) and a negative correlation between TPC and WIP (*r* = −0.716, and *r* = −0.788, respectively) were found. No significant correlation was found between parameters that describe the α-amylase activity and the starch properties, such as FN, APV, and FT (Table 5).

The bread yield (YB) ranged from 140% (bread from the flour RF2) to 156% (bread from the flour RF8). The YB was positively correlated with all viscosity parameters of the swelling curve test. Statistical analyses also showed a significant positive correlation between YB and AC, WA and WIP (*r* = 0.790, *r* = 0.713, and *r* = 0.758, respectively), and a significant negative correlation with Gr (*r* = −0.636, Table 5).

The crumb hardness is an important bread texture parameter. The lowest bread crumb hardness after one (BCH1) and four days’ (BCH4) storage of bread was observed in bread obtained from the flour RF4 (45.4 N and 59.6 N, respectively), whereas the highest values of these parameters were noted in bread from flour RF8 (103.5 N and 156.5 N, respectively). The increase in bread crumb hardness during storage of bread (IBCH) ranged from 14.2 N (bread from the flour RF4) to 63.1 N (bread from the flour RF2). No significant correlations were observed between BCH1, BCH4, and IBCH in the parameters that describe the α-amylase activity and starch properties, such as FN, APV, and FT. In the present study, bread from the flour RF3 had significantly higher values of FN, APV, and FT than the bread from the flour RF5 but had similar levels of BCH1, BCH4, and IBCH to the bread from the flour RF5. It was also noted that bread from rye flour RF6, which had optimal values of FN and FT, showed crumb characterized by one of the highest values of BCH1, BCH4, and IBCH. This finding indicates that for commercial rye flour, the FN and the amylograph properties are not sufficient parameters for predicting bread hardness and its increase during storage of bread. Further, it was observed that BCH1, BCH4, and IBCH were significantly and negatively correlated only with Gr and %WSP (Table 5). Generally, breads with lower hardness and lower increases in hardness during storage were obtained from rye flour samples with fine granulation and high %WSP. The crumb moisture (CM) ranged from 45.6% (bread from the flour RF9) to 51.4% (bread from the flour RF3). Only WSP had a significant impact on CM. The correlation between CM and WSP was *r* = 0.643 (Table 5). No correlation was observed between CM and WIP, which probably meant that other non-starch polysaccharides present in the insoluble fiber had an impact on the CM [6].

### 3.4. Principal Component Analysis (PCA)

**Figure 1:**
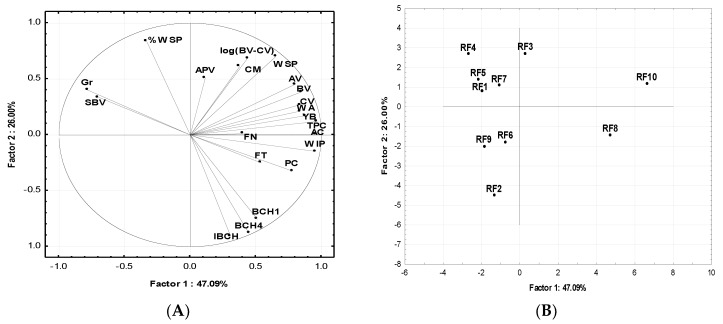
Principal component analysis: (**A**) Loading plot of PC1 and PC2 for the evaluated parameters of the tested rye flours and breads; (**B**) score plot of PC1 and PC2 for tested rye flours. SBV—specific bread volume, BCH1—bread crumb hardness one day after baking, BCH4—bread crumb hardness four days after baking, IBCH—increase of bread crumb hardness after 3 days storage of bread, CM—crumb moisture, PC—protein content, AC—ash content, Gr—flour granulation, FN—falling number, APV—amylograph peak viscosity, FT—final temperature of starch gelatinization, WA—water absorption, TPC—total pentosans content, WSP—water-soluble pentosans content, WIP—water-insoluble pentosans content, %WSP—percentage of the water -soluble pentosans content, AV—initial viscosity at 30 °C, BV—viscosity when the sample reached 42 °C, CV—viscosity after holding at 42 °C for 30 min, and log(BV − CV)—logarithmic decrease of viscosity at 42 °C.

The results of PCA shoved that only three out of nine principal components were found to be important, as their values were larger than 1, which is usually considered as the criterion for significance. Figure 1A shows that the first two principal components (PC1, PC2) explained 73.09% of the variation.

The first principal component (PC1) accounted for 47.09% of the variation and was the most related to AC, TPC, and WIP; all viscosities obtained from the swelling curve: AV, BV, and CV; and WA. PC1 was negatively correlated with Gr and SBV. The second component (PC2) explained 26% of the variation and was strongly positively related to the %WSP and WSP and negatively related to bread hardness and increase in hardness during storage of bread. 

Figure 1B shows that the two samples RF8 and RF10 were the most positively correlated with PC1. That finding indicates that these two rye flour samples had higher TPC, WSP, WIP, PC, and all viscosity parameters obtained from the swelling curve, than those of the other tested rye flours. Furthermore, the samples RF8 and RF10 had significantly lower %WSP, coarser granulation, and lower SBV than the other flour samples. As shown in Figure 1B, the flour sample RF2 was the most negatively correlated with PC2; that finding indicates that RF2 rye flour had significantly smaller WSP and %WSP, and a lower value of the logarithmic decrease of viscosity at 42 °C than the other tested rye flours. Compared to the other rye breads, the bread from rye flour RF2 had a higher hardness and a larger increase in bread hardness during storage.

## 4. Conclusions

1. The tested industrial wholemeal rye flours were significantly different in terms of all parameters that characterize their baking value. Many significant relationships were found between flour and bread properties.
2. The study revealed that for commercial rye flours, the falling number and the amylograph properties are not sufficient parameters for predicting the quality of wholemeal rye bread. To produce wholemeal rye bread with high volume, low crumb hardness, and a small increase in hardness during storage, it is recommend to use rye flour with fine granulation, low protein content, low total and water-insoluble pentosans content, and a high percentage of water-soluble pentosans content.
3. Bread with a higher volume was generally obtained from rye flour characterized by lower protein content, lower total pentosans content and water-insoluble pentosans content, and a higher percentage of water-soluble pentosans content. Further, flour granulation and the percentage of water-soluble pentosans content had a significant impact on bread crumb hardness, and its increase during storage of bread. Generally, breads with lower crumb hardness were obtained from flours with finer granulation and with a higher percentage of water-soluble pentosans content. Moreover, a positive correlation was found between crumb moisture and water-soluble pentosans content.

## Figures and Tables

**Table 1:** The basic quality parameters of tested rye flours.

Rye Flour	PC (Nx6.25) (% d.m.)	AC (% d.m.)	Gr (%)	WA (%)	FN (s)	APV (BU)	FT (°C)
RF1	10.4 ^b,^* ± 0.01	1.51 ^b^ ± 0.03	29.4 ^d^ ± 0.5	54.0 ^a^ ± 0.4	201 ^c^ ± 7	290 ^ab^ ± 14	71.0 ^cd^ ± 0.4
RF2	11.7 ^f^ ± 0.04	1.60 ^c^ ± 0.01	30.2 ^de^ ± 0.2	58.9 ^c^ ± 0.3	260 ^f^ ± 6	290 ^ab^ ± 7	74.5 ^f^ ± 0.0
RF3	10.6 ^bc^ ± 0.02	1.84 ^e^ ± 0.01	34.2 ^e^ ± 0.7	63.6 ^e^ ± 0.1	278 ^g^ ± 8	480 ^c^ ± 7	76.0 ^g^ ± 0.4
RF4	9.2 ^a^ ± 0.01	1.43 ^a^ ± 0.01	39.0 ^g^ ± 0.4	59.2 ^c^ ± 0.3	222 ^d^ ± 6	450 ^c^ ± 20	70.0 ^bc^ ± 0.4
RF5	10.6 ^bc^ ± 0.01	1.72 ^d^ ± 0.01	32.7 ^ef^ ± 0.7	60.4 ^d^ ± 0.1	180 ^b^ ± 4	290 ^ab^ ± 3	68.0 ^a^ ± 0.0
RF6	11.3 ^def^ ± 0.02	1.59 ^c^ ± 0.01	23.5 ^c^ ± 0.4	58.6 ^c^ ± 0.1	160 ^a^ ± 8	250 ^a^ ± 7	68.5 ^ab^ ± 0.4
RF7	11.0 ^cd^ ± 0.03	1.74 ^d^ ± 0.01	33.7 ^e^ ± 0.5	58.8 ^c^ ± 0.3	244 ^e^ ± 4	340 ^b^ ± 18	72.5 ^de^ ± 0.7
RF8	11.4 ^ef^ ± 0.02	2.34 ^f^ ± 0.02	13.6 ^a^ ± 0.1	65.3 ^f^ ± 0.4	254 ^ef^ ± 7	430 ^c^ ± 14	83.0 ^h^ ± 0.4
RF9	11.2 ^de^ ± 0.01	1.74 ^d^ ± 0.01	19.7 ^b^ ± 0.3	55.8 ^b^ ± 0.1	228 ^d^ ± 3	260 ^a^ ± 7	73.5 ^ef^ ± 0.7
RF10	14.0 ^g^ ± 0.05	2.42 ^f^ ± 0.03	12.5 ^a^ ± 0.4	66.6 ^g^ ± 0.2	247 ^ef^ ± 8	280 ^a^ ± 11	72.0 ^de^ ± 0.4

* The values designated by the different letter are significantly different (*n* = 3, *p* < 0.05); PC—protein content, AC—ash content, Gr—flour granulation, percentage of the flour fraction passed through sieve of 95 µm, WA—water absorption, FN—falling number, APV—amylograph peak viscosity, and FT—final temperature of starch gelatinization.

**Table 2:** The content of pentosans in tested rye flours and parameters of the swelling curves.

Rye Flour	TPC (% d.m.)	WSP (% d.m.)	WIP (% d.m.)	%WSP	AV (BU)	BV (BU)	CV (BU)	Log (BV − CV)
RF1	9.1 ^b^ ± 0.12	3.2 ^bc^ ± 0.08	5.4 ^a^ ± 0.02	35 ^cd^ ± 1	290 ^c^ ± 3	290 ^cd^ ± 7	280 ^d^ ± 7	15 ^b^ ± 1
RF2	9.5 ^cd^ ± 0.11	2.4 ^a^ ± 0.09	7.0 ^e^ ± 0.20	25 ^a^ ± 1	100 ^a^ ± 11	110 ^a^ ± 7	145 ^a^ ± 7	120 ^a^ ± 7
RF3	10.2 ^f^ ± 0.02	3.6 ^cd^ ± 0.16	6.5 ^cde^ ± 0.14	35 ^cd^ ± 2	335 ^d^ ± 7	325 ^e^ ± 7	255 ^d^ ± 7	105 ^d^ ± 3
RF4	9.4 ^bcd^ ± 0.11	3.2 ^bc^ ± 0.13	6.1 ^bc^ ± 0.24	34 ^cd^ ± 2	280 ^c^ ± 3	280 ^bc^ ± 3	250 ^cd^ ± 3	50 ^c^ ± 1
RF5	9.1 ^b^ ± 0.06	3.2 ^bc^ ± 0.11	5.9 ^abc^ ± 0.17	35 ^cd^ ± 1	290 ^c^ ± 14	270 ^bc^ ± 14	200 ^b^ ± 14	131 ^e^ ± 8
RF6	9.6 ^de^ ± 0.06	2.8 ^ab^ ± 0.05	6.8 ^de^ ± 0.01	29 ^ab^ ± 1	300 ^c^ ± 7	315 ^de^ ± 7	275 ^d^ ± 7	59 ^c^ ± 1
RF7	9.9 ^ef^ ± 0.11	3.7 ^d^ ± 0.10	6.2 ^bcd^ ± 0.21	37 ^d^ ± 2	270 ^c^ ± 7	260 ^bc^ ± 11	200 ^b^ ± 11	115 ^de^ ± 5
RF8	12.2 ^g^ ± 0.13	3.5 ^cd^ ± 0.11	9.7 ^f^ ± 0.24	29 ^ab^ ± 1	360 ^d^ ± 3	400 ^f^ ± 7	350 ^e^ ± 7	58 ^c^ ± 1
RF9	8.3 ^a^ ± 0.06	2.6 ^a^ ± 0.10	5.6 ^ab^ ± 0.09	31 ^bc^ ± 1	230 ^b^ ± 7	250 ^b^ ± 7	220 ^bc^ ± 7	56 ^c^ ± 2
RF10	13.4 ^h^ ± 0.02	3.9 ^d^ ± 0.06	9.2 ^f^ ± 0.05	29 ^ab^ ± 1	670 ^e^ ± 14	635 ^g^ ± 7	410 ^f^ ± 7	190 ^f^ ± 3

The values designated by the different letter are statistically significantly different (*n* = 3, *p* < 0.05). TPC—total pentosans content, WSP—water-soluble pentosans content, WIP—water-insoluble pentosans content, %WSP—percentage of the water-soluble pentosans, AV—initial viscosity at 30 °C, BV—viscosity of the sample reaching 42 °C, CV—viscosity after holding at 42 °C for 30 min, and log(BV − CV)—logarithmic decrease of viscosity at 42 °C.

**Table 3:** Correlation coefficients between parameters describing quality of rye flour.

Parame-Ter	AC	Gr	FN	APV	FT	WA	TPC	WSP	WIP	%WSP	AV	BV	CV	BV − CV
PC	0.731 **	−0.770 **	N	N	N	N	0.704 *	N	0.768 **	N	0.633 *	N	N	N
AC		−0.790 **	N	N	N	0.827 **	0.893 **	N	0.864 **	N	0.740 *	0.786 **	0.741 *	N
Gr			N	N	N	N	−0.638 *	N	−0.720 *	N	N	−0.639 *	−0.697 *	N
FN				N	0.721 *	N	N	N	N	N	N	N	N	N
APV					N	N	N	N	N	N	N	N	N	N
FT						N	N	N	N	N	N	N	N	N
WA							0.868 **	0.639 *	0.819 **	N	0.673 *	0.685 *	N	N
TPC								0.670 *	0.959 **	N	0.817 **	0.842 **	0.811 **	N
WSP									N	N	0.752 *	0.723 *	0.818 **	0.769 **
WIP										N	0.705 *	0.747 *	0.749 *	N
%WSP											N	N	N	N
AV												0.989 **	0.889 **	0.778 **
BV													0.939 **	0.741 *
CV														N

** Correlation is significant at *p* < 0.01 level, * Correlation is significant at *p* < 0.05 level, N not significant, PC protein content, AC ash content, Gr flour granulation, FN falling number, PV amylograph peak viscosity, WA water absorption, TPC total pentosans content, WSP water-soluble pentosans content, WIP water- insoluble pentosans content, %WSP percentage of the water-soluble pentosans content, AV initial viscosity at 30 °C, BV viscosity when the sample reached 42 °C, CV viscosity after holding at 42 °C for 30 min, and log(BV − CV)—logarithmic decrease of viscosity at 42 °C.

**Table 4:** The quality parameters of rye breads.

Rye Flour	SBV (cm^3^/100 g)	YB (%)	BCH1 (N)	BCH4 (N)	IBCH (N)	CM (%)
RF1	172 ^c^ ± 1	145 ^abc^ ± 1	60.8 ^c^ ± 0.4	89.8 ^d^ ± 0.1	29.0 ^d^ ± 0.3	50.4 ^cde^ ± 0.2
RF2	148 ^a^ ± 3	140 ^a^ ± 3	90.2 ^f^ ± 0.1	153.3 ^i^ ± 0.7	63.1 ^h^ ± 0.6	48.5 ^cd^ ± 0.1
RF3	179 ^cd^ ± 1	150 ^cd^ ± 1	54.9 ^b^ ± 0.6	77.5 ^b^ ± 0.8	22.6 ^c^ ± 0.3	51.4 ^e^ ± 0.1
RF4	186 ^de^ ± 1	143 ^abc^ ± 1	45.4 ^a^ ± 0.3	59.6 ^a^ ± 0.7	14.2 ^a^ ± 0.4	50.2 ^cde^ ± 0.3
RF5	190 ^e^ ± 3	142 ^ab^ ± 2	54.9 ^b^ ± 0.7	84.4 ^c^ ± 0.8	29.5 ^d^ ± 0,1	49.5 ^cde^ ± 0.1
RF6	187 ^e^ ± 1	149 ^bcd^ ± 3	80.9 ^d^ ± 0.1	132.0 ^h^ ± 0.3	51.1 ^f^ ± 0.1	46.0 ^ab^ ± 0.2
RF7	177 ^c^ ± 1	145 ^abc^ ± 1	82.4 ^de^ ± 0.8	102.0 ^e^ ± 1.4	19.6 ^b^ ± 0.6	47.9 ^abc^ ± 0.6
RF8	156 ^b^ ± 1	156 ^d^ ± 3	103.5 ^g^ ± 0.4	156.5 ^i^ ± 0.7	53.0 ^g^ ± 0.3	49.8 ^cde^ ± 1.2
RF9	191 ^e^ ± 3	143 ^abc^ ± 1	89.0 ^f^ ± 0.6	128.0 ^g^ ± 0.9	39.0 ^e^ ± 0.4	45.6 ^a^ ± 1.1
RF10	146 ^a^ ± 2	154 ^d^ ± 1	83.4 ^e^ ± 0.6	121.2 ^f^ ± 1.3	37.8 ^e^ ± 0.7	51.0 ^de^ ± 0.7

The values designated by the different letters are statistically significantly different (*n* = 3, *p* < 0.05); SBV—specific bread volume, YB—bread yield, BCH1—bread crumb hardness one day after baking, BCH4—bread crumb hardness four days after baking, IBCH—increase of bread crumb hardness after 3 days storage of bread, and CM—crumb moisture.

**Table 5:** Correlation coefficients between the rye flour and bread properties.

Parameters	SBV	YB	BCH1	BCH4	IBCH	CM
PC	−0.689 *	N	N	N	N	N
AC	N	0.790 **	N	N	N	N
Gr	N	−0.636 *	−0.751 *	−0.720 *	−0.639 *	N
FN	N	N	N	N	N	N
APV	N	N	N	N	N	N
FT	N	N	N	N	N	N
WA	N	0.713 *	N	N	N	N
TPC	−0.716 *	0.809 **	N	N	N	N
WSP	N	N	N	N	N	0.643 *
WIP	−0.788 **	0.758 *	N	N	N	N
%WSP	0.641 *	N	−0.648 *	−0.810 **	−0.892 **	N
AV	N	0.716 *	N	N	N	N
BV	N	0.784 **	N	N	N	N
CV	N	0.850 **	N	N	N	N
log(BV − CV)	N	N	N	N	N	N

** Correlation is significant at *p* < 0.01 level, * Correlation is significant at *p* < 0.05 level, N—not significant; PC—protein content, AC—ash content, Gr—flour granulation, FN—falling number, APV—amylograph peak viscosity, WA—water absorption, TPC—total pentosans content, WSP—water-soluble pentosans content, WIP—water-insoluble pentosans content, %WSP—percentage of the water-soluble pentosans content, AV—initial viscosity at 30 °C, BV—viscosity when the sample reached 42 °C, CV—viscosity after holding at 42 °C for 30 min, log(BV − CV) logarithmic decrease of viscosity at 42 °C, SBV—specific bread volume, BY—bread yield, BCH1—bread crumb hardness one day after baking, BCH4—bread crumb hardness four days after baking, IBCH—increase of bread crumb hardness after 3 days storage of bread, and CM—crumb moisture.
